# Ageism and lookism as stereotypes of health disparity in intensive care units in Iran: a critical ethnography

**DOI:** 10.1186/s12939-024-02180-w

**Published:** 2024-06-03

**Authors:** Sajad Yarahmadi, Mohsen Soleimani, Mohammad Gholami, Ali Fakhr-Movahedi, Seyed Mohsen Saeidi Madani

**Affiliations:** 1 Student Research Committee, Semnan University of Medical Sciences, Semnan, Iran; 2 Social Determinants of Health Research Center, School of Nursing and Midwifery, Lorestan University of Medical Sciences, Khorramabad, Iran; 3 Nursing Care Research Center, School of Nursing and Midwifery, Semnan University of Medical Sciences, Semnan, Iran; 4 Department of Social Sciences, Yazd University, Yazd, Iran

**Keywords:** Ageism, Critical ethnography, Health disparity, Lookism, Intensive care unit

## Abstract

**Background:**

The intensive care unit presents structural complexities, and the prevailing power imbalance between patients and staff can lead to health disparities. Hence, unveiling the underlying factors that give rise to and reinforce these disparities can contribute to their prevention. This study aims to shed light on the stereotypes linked to ageism and lookism, which perpetuate health disparities within the intensive care unit setting in Iran.

**Methods:**

This critical ethnographic study employed Carsepkan’s approach and was carried out in intensive care units in the west of Iran from 2022 to 2023. The data collection and analysis were conducted through three interconnected stages. In the initial stage, more than 300 h of observations were made at the research site. In the subsequent stage, a horizon analysis was performed. Conversations with 14 informants were conducted in the final stage to enrich the dataset further. Then the analysis process was carried out as in the previous step to uncover an implicit culture of health disparity. To verify the validity and reliability of the study, credibility, conformability, dependability, and transferability were all taken into account.

**Findings:**

The ageism and lookism stereotypes emerged from seven main themes; youth-centric; negative ageism; age-friendliness; age-related priority; centered care for pediatric patients and families; appearance-centeredness; and a contradiction between belief and behavior.

**Conclusion:**

This critical study showed that ageism and lookism stereotypes permeated the intensive care unit’s culture. These stereotypes have the potential to influence equality dynamics, as well as to foster and support health disparity in the intensive care unit.

## Background

The Intensive Care Unit (ICU) is a clinical setting with a higher mortality rate and acuity, which might exacerbate the effect of disparities on patients [1]. Also, the available literature demonstrates that there are evident health disparities among patients who are critically ill, and the ICU can be a potential setting for health disparities [2–4].

The four principles of biomedical ethics proposed by Beauchamp and Childress—autonomy, beneficence, non-maleficence, and justice—have formed the foundation of medical ethics [5]. The principle of justice asserts that individuals should have fair and equitable access to healthcare, encompassing how healthcare resources are allocated and accessed, a concept known as distributive justice. Most societies are constrained by factors such as finances, politics, and access to services [5, 6]. This principle is crucial in addressing health disparities, which refer to specific types of health differences influenced by policies, creating gaps where socially disadvantaged groups experience worse health outcomes or higher risks compared to advantaged social groups [7]. Healthy People 2030 defines a health disparity as “a particular type of health difference linked with social, economic, and/or environmental disadvantage,” adversely affecting groups of people who have systematically experienced more significant obstacles to health” [8].

Like many countries, Iran grapples with disparities across various social determinants of health-related issues [9]. A cross-sectional study conducted in Iran revealed a significant disparity in the health outcomes of COVID-19-infected patients between urban and suburban areas [10]. Additionally, findings from qualitative studies have illuminated certain dimensions of health disparity that have often been overlooked in quantitative research [11, 12]. While the Iranian constitution and other upstream policies strongly support health equity and equitable access to healthcare services, Iran still requires a comprehensive national examination of the state of health disparity despite the availability of numerous theoretical frameworks, measurement methods, and approaches to address this issue. Nonetheless, achieving these goals remains highly challenging [13].

Studies show that health disparities occur due to various stereotypes such as ageism and lookism [14, 15]. “Ageism” was a term first introduced by Butler in the 1960s and is often used to describe negative attitudes and behaviors toward individuals based on their age, regardless of whether they are considered “old” or “young“ [16, 17]. This term highlights the harmful impact of age-related biases and the resulting societal disparities. Ageism can manifest in different aspects of life, including the workplace, healthcare, and social settings. It can substantially affect individuals’ well-being and quality of life [16].

Ageism encompasses different forms, including positive and negative ageism, which refers to stereotyping populations based on favorable or unfavorable age-related connotations. Other forms of ageism are implicit and explicit and personal or institutional. Ageism can take many other forms and negatively affect individuals and society if not addressed [18]. Evidence shows a clear link between ageism and health disparities [14, 19, 20]. A review study showed that ageism can affect health outcomes [14]. The results of Mehta et al.‘s study showed that the additive scale of the excess death risks posed by key socio-demographic and behavioral risk factors increases with age [19]. The results of Dudley and Taek Lee’s study in China showed that the peak mortality of COVID-19 was in the age group of 50–59 years, while the peak death rate was in the age group of 80 years and above. Also, this study showed that in South Korea, the peak morbidity was related to the age group of 20–29 years, while the peak mortality rate was related to the age group above 80 years [20].

Considering that ageism is both common and associated with poor health outcomes, as well as the varied patterns of ageism observed in diverse societies, it is imperative to identify its occurrence and associated areas. This identification is crucial for developing targeted interventions aimed at mitigating adverse health outcomes linked to ageism and related disparities [14]. Recognizing this gap emphasizes the need for rigorous qualitative studies to explore ageism’s complex dynamics and its intersections with health disparities. Conducting such studies is crucial for capturing nuanced age-related discrimination and informing evidence-based interventions and policy frameworks, enabling health policymakers to design tailored interventions that promote greater equity and inclusivity in healthcare settings.

Lookism pertains to bias rooted in physical appearance, whether attractive or not [21]. Physical appearance impacts almost every aspect of our lives, such as personal relationships, self-confidence, job prospects, and financial rewards [22]. Facial attractiveness, particularly physical features that signify youth, plays a significant role in human interaction and is often perceived as advantageous. Evidence shows that other people’s judgments of one’s appearance can shape self-perceptions of positive or negative qualities. The social benefits of an attractive facial appearance are widespread and notable [23]. The characteristic attractiveness of a person’s appearance elicits reactions from others, and the impact of an individual’s physical attractiveness on initial impressions has been extensively studied. Stereotyping based on appearance is prevalent among various age groups, which contradicts the idea that judgments should not be influenced by personal characteristics such as physical attractiveness [24].

Many empirical studies underline that lookism is a widespread and systematic manifestation of social prejudice [15, 21]. However, lookism has received little attention, despite emphasizing ethical philosophy. This discrepancy becomes especially noticeable when contrasted with other types of discrimination, like those based on race or gender, which have been prominently explored as research topics in the context of injustice [21].

A study states that attractive people are perceived to possess positive attributes, and these traits are activated rapidly and unconsciously when we see their faces. Attractive individuals are often viewed as more friendly, generous, and intelligent than their less attractive counterparts [23]. This highlights the disparity of being prejudiced against someone based on their looks and features. A cohort study in South Korea showed that perceived appearance discrimination is associated with the health of adults appearance discrimination [15]. Also, another study contends that lookism is linked to diverse types of epistemic unfairness [21]. To address these gaps in understanding, there is a critical need to conduct rigorous studies that delve into the complexities of lookism in ICU patients and their families, shedding light on its pervasive influence on societal attitudes and health outcomes.

This study was conducted to uncover the cultural structures of power that construct and bring into the flow a health disparity in the ICU in Iran. The study identified ageism and lookism stereotypes as part of the prevailing culture in the research site, and the current study aims to describe and explain this culture.

## Methods

### Design

The study utilized an ethnographic approach to comprehensively understand the factors influencing people’s experiences when interacting with the healthcare system. Additionally, the study aimed to uncover the power dynamics that contributed to health disparities in ICUs and employed a critical ethnography method [25]. Critical research recognizes that power imbalances and oppressive forces exist in all forms of human interaction and that systematical structures that have the potential to be oppressive may impede the provision of care centered around the individual’s needs and preferences. Analyzing culture from the perspective of power, privilege, and authority allows one to uncover unfair and unjust systems and identify whose voices are amplified and suppressed [26].

One of the most impactful methods in critical ethnography is the Carsepkan approach (1996). The approach taken by Carspecken focuses on how social disparity and social structure are replicated through conventional practices. The foundation of Carspecken’s critical ethnographic approach is that critical ethnography is a form of social activism in which “critics” conduct research that exposes preexisting systems of dominance, hidden assumptions, and ideologies to redefine social situations and power dynamics. Critical ethnographic research can advance social theory, expose systematic inequities rather than merely report social reality, and contribute to people’s well-being [27]. Therefore, the Carsepkan approach was used to conduct this study. This approach extends beyond merely describing the theoretical framework and imparts data collection and analysis strategies. The Carsepkan approach has also recently gained recognition in nursing research [27–29]. Among some of these studies, we can refer to the study by Boozerzad et al., who investigated the cultural safety of patients [30], and the study by Shirani Bidabadi et al., who explored patient dignity in the intensive care unit [31].

### Research site

This study was conducted from August 2022 to August 2023 in the ICUs of Shohadaye Ashayer Hospital, which serves as a teaching, referral, and trauma center with 300 beds and six ICUs. The hospital is located in the center of Lorestan province, in the western region of Iran, known for its substantial cultural diversity. The ICUs at this hospital cater to a range of patients, including those with trauma, infectious diseases, and surgical needs. On average, there are 30 patients in the ICUs at any given time, with each patient staying approximately 14 days in the hospital. The ICUs are supported by a diverse and relative number of physicians, along with six head nurses, 75 nurses, 19 attendants, and six secretaries. Each staff member works an average of 25 six-hour shifts per month, with night shifts counted as two shifts.

### Informants

The study’s informants included individuals who have a role in creating and shaping the study setting culture, such as nurses, physicians, patients, their families, attendants, etc.

### Data collection and analysis

According to Carspecken, critical ethnography involves three preliminary and five main stages. Preliminary stages include:

- Creating a list of research questions: Carspecken advised ethnographers not to focus solely on one research question but instead to prepare a comprehensive and flexible list of questions related to the main research question [27]. This study prepared a list of questions that could help explain the culture of disparity in the ICU.
- A list of specific items for investigation: At this stage, the situations that enabled the research team to answer the questions from the previous stage were identified [27].
- The reflective journal to determine the researcher’s value orientations was recorded before beginning the main stages to minimize potential biases [27]. Table 1 provides examples of the lists prepared in the preliminary stage.

**Table 1:** Examples of the lists prepared in the preliminary stage

First list	Second list	Reflective Journal
How does health disparity manifest in the ICU?	Participate in formal and informal interviews with key informants.	Everyone should reach their full health potential.
Under what circumstances does health disparity take place in the ICU?	Observing the study’s site, informants’ behavior and interactions, and how they care for patients.	Moral knowledge should challenge empirical knowledge, and empirical knowledge should be presented as moral knowledge.
What factors contribute to or mitigate health disparities in the ICU?	Field journals should include any additional notes and memos during the data collection.	Care issues should be viewed through a social lens.

The main stages include:

- The first stage involved creating a primary record by observing the intricate interactions among all individuals involved in creating and affecting culture. The data collected during this stage were considered monological, as there was minimal dialogue between the observer and the observed informants [27]. The focus was observing all actions related to the health disparities in care, treatment, and other services. Additionally, various resources were observed, including documents, guidelines, policies, physical space, and equipment. The researcher’s primary role in data collection was observer-participant. Monological data were generated through over 300 h of presence and observation at the research site, which primarily focused on an ICU but also encompassed other ICU departments and hospital units that could influence the formation of arena culture in a decentralized manner. Observations were generally conducted during three work shifts—morning, noon, and night—with a primary focus on the morning shift due to higher levels of interaction.
- During the second stage, the primary data was analyzed using various techniques to identify patterns of interaction, power dynamics, roles, and other relevant factors. The analysis was referred to as “reconstructive” because it aimed to reveal cultural themes and systemic factors that the people providing the data may not have explicitly expressed, and there was always some ambiguity or uncertainty. This stage involved three interrelated activities; low-level coding, initial meaning reconstruction, and operational horizon analysis. Low-level coding involves assigning codes to the data closely related to the raw data. Reconstruction of the initial meaning applied a mental analysis or hermeneutic that guided the interpretation of the data. Finally, the analysis of the operational horizon included subjective, objective, and normative-evaluative claims that helped to create more abstract and high-level codes [27].
- In the third stage, dialogic data was produced by engaging in lengthy conversations with informants using informal conversation and interviews. This aimed to give informants a voice in the research process instead of solely relying on the researcher’s perspective. Data was generated with the informants through dialogue rather than collected monolocical data about them. This stage was seen as a way to democratize research, per Carsepeken. An interview guide was prepared based on the research questions and the previous stage data, and semi-structured interviews were also conducted alongside informal conversations. The information obtained during this stage often challenged the data collected in the previous stages, leading to further attention and revised analyses [27]. The dialogic data were mainly generated in one of the educational spaces within the hospital. The duration of the interviews ranged from 30 to 60 min. Table 2 presents information related to the informants.

**Table 2:** Information related to the informants

Informants variables	Values
Age*	41.2 (9.1)
Years in the workforce for staff* (years)	11.7 (8.3)
Duration of ICU stay for patient and family* (days)	14 (3.3)
Gender - Female - Male	9 5
Role - Nurse - Patient’s family - Patient - Attendant - Physician - Hospital official	8 2 1 1 1 1
Marital status - Single - Married - Widow	5 8 1
Education - Below a bachelor’s degree - Bachelors and Masters - Doctorate and above	4 9 1

*Mean (Standard Deviation)

In the monological and dialogical stages, tone and body language were considered. Observations and informal conversations were recorded immediately, and quick field notes were made. All semi-structured interviews were recorded on tape after obtaining the informants’ consent and then transcribed.

The main stages include:

(d) Discovering system relations, and (e) using system relations to explain findings. However, we followed Carspecken’s recommendation for novice researchers and only implemented the first three stages [27].

### Reflexivity

The researcher constantly thought about how his situation, history, experiences, and biases might influence the research process. It has also reviewed its position at each stage. This required understanding the deep values and beliefs of the researcher [27].

### Rigor

Carsepkan suggested that researchers consider a combination of interviews, observations, and documents to reduce bias through triangulation [17, 19]. In addition, he suggested the Lincoln and Guba validation methods used to certify monological and dialogical data [27]. Lincoln and Guba (1994) proposed credibility, dependability, transferability, and confirmability as criteria of scientific trustworthiness in qualitative research, which were used in this study [32]. A series of measures were taken to ensure the accuracy and validity of the findings, including using a flexible observation schedule, memo writing, and field notes of observations conducted at different times. Peer review was conducted weekly. The audit trail and how to decide at each stage for collecting information were clear. The researcher was on the site for a long time and was engaged in the interactions as an observer-participant. An informant was asked to review the analyses to accommodate cultural sensitivities to ensure accurate interpretations. A descriptive summary of the findings was also shared with informants [33].

## Findings

Two stereotypes, seven themes, and 19 high-level codes have emerged in this critical ethnographic study (Table 3).

**Table 3:** Summary of themes and high-level codes

Stereotypes	Themes	High level codes
Ageism	Youth-centric	Social preferences
		Top focus
		Affectionate communication
	Negative ageism	Negative attitudes toward the elderly
		Less sensitivity and poorer care
	Age friendliness	Respect for elders
		Positive ageism
	Age-related priority	Prioritization
		Age affinity
	Centered care for pediatric patients and families	Empathy with the family
		Increased attention
		Compassion
Lookism	appearance-centeredness	Weight-based discrimination
		Hygiene-based discrimination
		Beauty worship
		Appearance-conscious
		Effective communication
		Memorable face
	A contradiction between belief and behavior	A contradiction between belief and behavior

### Ageism

This stereotype emerged from five themes: youth-centric, negative ageism, age-friendliness, age-related priority, and centered care for pediatric patients and families.

### Youth-centric

The results of this study showed that one of the dominant cultures in ICUs is youth-centered, which emerged from the induction of three high-level codes: “social preferences,” “top focus,” and “affectionate communication.” Within this culture, young individuals are often provided with better care and attention. However, it is essential to acknowledge that all patients should receive care based on the potential for their health to reach that level.

#### Social preferences

Patients’ families, hospital officials, the hospital system, and society prefer better care and treatment for young patients. The viewpoint is that they should have more opportunities to live. *The family of the elderly patient protested to the nurse, questioning why another patient’s family was permitted to enter the ward and be engaged in their patient’s care while they were not given permission. The nurse responded with a serious expression, “Their patient is young.” This response convinced the family of the elderly patient, and they did not continue their protest.* (Observation, ICU entrance corridor)

*“Sometimes I see the head nurse or doctors tell us, “Hurry up and work faster; the patient is young!”* (Interview, Informant 1).

#### Top focus

The staff shows more focus and attention when providing care for young patients. Typically, staff dedicate more time and energy to care for young patients, resulting in higher quality and accurate work. Additionally, they utilize various medical consultations specific to them and employ more skilled nurses to ensure proper care.

“*When I got a young patient, I spend most of my time directly takin’ care of ‘em, to the point that I gotta write my nursing report in the next shift.*” (Interview, Informant 2).

#### Affectionate communication

In the ICU, more time is spent communicating with young patients. Also, the staff strives to create a less formal atmosphere and establish a more affectionate relationship with the patients.

*The nurse was doing cupping manner chest physiotherapy on a young patient and laughingly said to the young patient, I am hitting you, the patient also laughed and said to calm down.* (Observation, bedside)

“*I’m tryna build a closer, warmer relationship with ‘em.*” (Interview, Informant 2).

### Negative ageism

Negative ageism can lead to older patients being perceived as less deserving of nursing care, and medical interventions or less likely to recover. This bias can result in age-based discrimination in treatment decisions and resource allocation, potentially compromising the quality of care provided to elderly patients. Negative ageism emerged from high-level codes, including a “negative attitude toward the elderly” and “less sensitivity and poorer care.”

#### Negative attitudes toward the elderly

Negative attitudes toward the elderly have been observed, leading to potential biases in their care and treatment. These attitudes may result in less attention toward the needs of older patients, impacting the quality of care provided to them.

*The nurse said to the other: “He has lived his life; we don’t need to be too delicate about it.”* (Observation, nurse station).

#### Less sensitivity and poorer care

The older patients receive less sensitivity, reduced evaluation, decreased attention, less meticulousness in tasks, and staff presence is less frequent at their bedside. Additionally, their beds may be placed in marginal positions in the section.

*An elderly patient with a tracheal tube gestured for the surgical resident to approach him as if he had something to be concerned about. However, the resident, with an expressionless face, did not respond to the patient, despite witnessing the patient’s evident struggle to get his attention.* (Observation, bedside)

“*We’re less sensitive about that patient, and we don’t go to check on him as often.*” (Interview, Informant 3).

### Age friendliness

Age friendliness involves providing care for older patients, which is deeply ingrained in the culture of Iranian society, emphasizing respect for the elderly. However, traces of this societal culture have extended to the ICU. This culture promotes respect, improved communication, and addressing psychological needs for the elderly, but it often lacks focus on physiological care. This theme emerged from two high-level codes; “respect for elders” and “positive ageism.”

#### Respect for elders

The family’s interest in the elderly patient, the particular position of the elderly in the family, and the staff’s sense of respect for the elderly affect the staff’s caring behavior.

“*There was this 80-year-old lady who was our patient, and her family was very concerned about her. They were always at the hospital because they respected her a lot. Her condition was such that we might have been able to keep her stable with more care, so we were trying to put in more effort for her.*” (Interview, Informant 3).

#### Positive ageism

There is a cultural current of positive ageism in the ICU, where some elderly receive better care for communication and psychosocial needs. However, this type of care seems to be primarily directed toward elderly patients who exhibit cheerful, beautiful, articulate, and affectionate characteristics or remind the staff of elderly family members. Additionally, those elderly patients who evoke pity and compassion from the staff may receive better psychosocial care.

*The elderly patient said to the attendant, “I’m bored! My children came to visit me late today.” The attendant said cheerfully, “I’ll play music on my mobile cell phone to lift your spirits!”* (Observation, bedside).

“*I had a cute little elderly patient that I really liked, and I would spend way more time with them*.” (Interview, Informant 4).

### Age-related priority

Some patients in various age groups are given higher priority for optimal care. This theme was derived from two high-level codes: “prioritization” and “age affinity.”

#### Prioritization

To provide quality care, the staff prioritizes equipment and time spent on patients based on age, giving young patients a higher priority.

“*Once, we had to admit a critically ill patient, but all ICU beds in the hospital were blocked. We had to decide to transfer one of the patients to the general ward. Most of our patients were young, and we had to transfer one of the older patients.”* (Interview, Informant 5).

#### Age affinity

Patients who are similar in age are better understood by staff, which can provide better care. Additionally, since the majority of the staff at the research site are young people, young patients may often receive additional benefits.

*Experienced nurses said*: “*I understand patients of my age better; for example, when a patient says their back hurts, I can relate to it better because I’ve experienced it myself.*” *The younger nurse also said*: “*When I see a patient of the same age and generation as me, I try to get closer to them and provide more care and attention.*” (Observation, nurse station).

### Centered care for pediatric patients and families

This theme which revolves around the focused attention and care provided to child patients and their families is a prevailing cultural trend in the ICU. It was derived from three high-level codes: “empathy with the family,” “increased attention,” and “compassion.”

#### Empathy with the family

The staff empathized and identified with and related to the families of the pediatric patients.

“*I imagine myself in the position of that family whose child is hospitalized in the ICU bed.”* (Interview, Informant 1).

#### Increased attention

The staff pays more attention and cares for pediatric patients, trying to reduce their suffering.

“*Because the nurses knew my kid couldn’t handle it, they were always trying to ease his pain.*” (Interview, Informant 6).

#### Compassion

In the research site, the staff provides compassionate care for pediatric patients, and a flow of emotions influences their care.

*The nurse gently stroked the hair of a 13-year-old girl with a low consciousness level and sang softly to her.* (Observation, bedside)

“*I’ve been working here for a long time, but this thing still hasn’t become normal for me, and sometimes I even cry for the pediatric patients*.” (Interview, Informant 4).

### Lookism

This stereotype emerged from two themes: appearance-centeredness and a contradiction between belief and behavior.

### Appearance-centeredness

Appearance-centeredness in the ICU refers to bias and discrimination based on patients’ physical appearance. This phenomenon is rooted in the high-level codes identified in the study, such as “weight-based discrimination,” “hygiene-based discrimination,” and “beauty worship.” In the ICU, there may be a tendency to prioritize patients perceived as beautiful or more pleasing, leading to potential disparities in the quality of care. Furthermore, staff may exhibit a bias in their communication and attention or “effective communication,” focusing more on patients with “memorable faces” or “appearance-conscious” individuals.

#### Weight-based discrimination

Patients with higher weights experience discrimination regarding care, treatment, or even diagnostic tests such as CT scans. The existing equipment is typically designed for normal-weight patients and may not be suitable for higher weights or larger sizes.

*A large and obese patient was placed in a bed that was too small for him in such a way that the staff could not change the patient’s position. One of the staff said, “I hate working with heavy patients.”* (Observation, bedside).

#### Hygiene-based discrimination

Intensive care unit staff react to the unclean appearance of patients and tend to avoid or distance themselves from unhygienic patients.

*“We had a dirty, smelly patient, and I tried not to stare at them or get too close.”* (Interview, Informant 4).

#### Beauty worship

Patients who possess attractive looks, consciously or unconsciously, tend to receive more attention from the staff. Additionally, they tend to leave a stronger impression, and their care details are often remembered. Staff members generally engage more effectively with them and sometimes establish a more pleasant communication.

*The patient’s nurse gently touched an attractive patient’s face while saying, “You look so beautiful!”* (Observation, bedside).

#### Appearance-conscious

Staff pay more attention to patients who care about their appearance than others.

“*As soon as we extubated the patient, she immediately said, “Could you please fetch my makeup accessories?” Well, you know, a patient who takes care of their appearance differs from the others. It means the little things matter to them. It’s like they pay attention to our behavior as much as they do to themselves.”* (Interview, Informant 7).

#### Effective communication

Patients or their families with a well-groomed appearance are treated more respectfully. Staff spend more time communicating with them, patiently explaining the situation, and providing necessary instructions.

*A young woman with a beautiful and well-groomed appearance came to the ICU to visit her mother. The nurses treated her respectfully, and the surgeon spent significant time explaining the patient’s condition.* (Observation, ICU hall)

#### Memorable face

Distinct and unique faces, whether beautiful or not, leave a lasting impression on the staff’s minds, and this remembrance can help minimize errors in their care.

“*We don’t forget distinctive faces, but sometimes we mix up ordinary faces*.” (Interview, Informant 8).

### A contradiction between belief and behavior

During monological and dialogical stages, the researcher noticed that ICU staff react to patients’ appearances. However, during the dialogical stage, some informants expressed their belief in the equal treatment and care of all patients regardless of their appearance. However, whether consciously or unconsciously, this value of patient equality was not consistently reflected in their actual action.

*The nurse (Informant 9) lifted the left hand of the unconscious patient to attach the electrocardiogram leads. Upon noticing the patient’s armpit was hairy, she scowled and let go of the patient’s hand from above.* (Observation, bedside)

“*I might unconsciously respond to patients’ appearances, yet these reactions are unintentional and not inherent in my deliberate caregiving behavior.”* (Interview, Informant 9).

*The nurse (Informant 10) told her colleague that when we have a patient who is so beautiful, I enjoy taking care of her and try to spend more time at her bedside.* (Observation, nurse station)

“*The appearance of patients usually doesn’t really impact how I behave or perform. Anyway, my approach to this issue as a nurse is different, and I’ve accepted it as part of my job.*” (Interview, Informant 10).

## Discussion

The findings of this critical ethnographic study revealed that ageism and lookism stereotypes are deeply ingrained within the care culture of the ICU. These stereotypes have the potential to influence parity dynamics, as well as to foster and support health disparity in the ICU.

One of the prominent themes highlighted in this study is youth-centricity, where younger patients are given more attention and care than older. This bias toward youth can have significant implications for the quality of care received by older patients, potentially leading to age-related disparities in healthcare outcomes. Another matter observed is an age-related priority, which suggests that specific medical interventions, treatments, or care may be prioritized based on a patient’s age rather than their health needs. This bias can undermine patient-centered care and equitable access to healthcare services.

The human lives contending for priority in a scarce situation are not segmented. Some individuals would have a higher value than others if human lives were measured in terms of life years at the point of evaluation, offering some people an unfair advantage. That would be prejudice. If choices were made only based on anticipated remaining life years, some patients would be unfairly given an advantage. Because the young have a higher life expectancy than the old (all other factors being equal), saving the most years of life is equivalent to preferring the young. This contradicts the fundamental principle of treating all human beings equitably [34, 35]. However, the statement in the Guidelines that the allocation “will favor those with the most number of life-years” does exist [36]. This statement is based on the assumption that, in usual circumstances, allocation is directed toward individuals with a higher life expectancy. Nevertheless, in the ICU, the majority of patients are in critical condition, and the patient’s status dictates which individual is more likely to survive.

Negative ageism also indicated discriminatory attitudes or beliefs toward older adults within ICU settings. These negative perceptions can contribute to suboptimal care and exacerbate health disparities among older patients. A qualitative study’s findings in Iran revealed that older patients perceive age discrimination by healthcare staff and disparities in the provided care in hospitals [37]. Also. the results of a cross-sectional study indicate that ICU nurses have a low level of knowledge and a negative attitude toward ageism [38].

On a positive note, age-friendliness emerged as a theme that promotes inclusive and supportive environments for some older patients within ICUs. This highlights the importance of creating spaces where older adults feel respected and valued and receive appropriate care tailored to their needs. This study demonstrated that psychosocial and communicative care for certain elderly patients, especially those who can communicate effectively, is performed well. This approach is rooted in the societal culture of Iran. However, a divergence in behavior becomes evident within the ICU environment when delivering physical and physiological care to older patients. As previously mentioned, a tendency exists to prioritize young patients for physiological and physical care. The findings of a study indicated that communication and healthcare services were perceived as lacking age-friendliness in older people. At the same time, social engagement, dignity, and inclusiveness were the most impacted aspects [39]. Utilizing the WHO framework presents a promising approach to evaluating healthcare policies about age-friendliness [40], and we recommend its ongoing refinement for this specific context.

The study also revealed a theme centered on pediatric patients and families, emphasizing this population’s need for specialized care and attention. Recognizing these specific needs can help enhance pediatrics receiving intensive care treatment outcomes. The research is conducted within the adult trauma center setting. However, pediatric patients may also be admitted to ICUs under critical conditions. The staff considers them distinct patients and offers care for patients and their families. The findings of a scoping review study revealed that respect, dignity, communication, participation, and collaboration were the foundational concepts of family-centered care that surfaced in pediatric ICUs [41]. The findings of this study corroborate the emerging theme in the current research.

Appearance-centeredness involves biases based on individuals’ physical attributes or appearances, which can impact how healthcare providers treat them. Addressing this issue is essential for promoting fairness and parity within healthcare settings. According to a study, beauty bias permeates all facets of social perception and interpersonal interaction, including hiring decisions, academic assessments, and outcomes in the criminal justice system [42]. The current study also expresses that this strain might manifest in healthcare settings such as the ICU. The findings of another study indicated that attractive people experience a better life than others [43]. Our study’s results reveal that this advantage for attractive individuals extends even to the ICU, where attractive patients seem to have better experiences.

Physical appearance discrimination in healthcare was another significant theme observed within ICUs. This form of lookism can lead to biased treatment based on an individual’s physical appearance rather than their medical condition or needs. These processes become even more deceptive since their legitimacy is less scrutinized compared to situations involving a criterion shared by a collective, such as race or gender [44]. The results of a critical ethnographic study showed that focusing solely on the patient’s body is a reductionist approach that contradicts the holistic approach [31].

One of the themes identified in this study was “A contradiction between belief and behavior” among staff. The recognition and acceptance of one’s professional role and adherence to its fundamental principles, such as refraining from differentiation or judgment of patients based on their appearances, could contribute to growing a culture that requires further reinforcement. It is critical to recognize that staff did not consciously decide how they would behave. The cultural atmosphere of the ICU affected the staff’s behaviors. Staff can decrease health disparities by raising awareness and engaging in critical self-reflection. The findings of an ethnographic study revealed a gap between the values and actions of healthcare workers in upholding the dignity of ICU patients [31].

This paper highlights important issues related to ageism and lookism within ICUs by examining these stereotypes through a critical ethnographic lens. We can strive toward more inclusive and patient-centered care practices by challenging these biases and reducing health disparities in healthcare delivery regardless of age or physical appearance. It is recommended to emphasize this study’s outcomes to offer solutions to alleviate health disparities in ICUs. Furthermore, the conclusions of this research can be extrapolated to diverse domains within nursing and medical fields, encompassing research, education, and practice, particularly within society. Given the study’s specific context, conducting additional qualitative investigations in alternative settings would prove beneficial in addressing health disparities comprehensively.

The incapability of written language to adequately describe interactions based on nonverbal cues like posture, gesture, and facial expressions is one of the limitations of this study. Additionally, the limitation on using additional words hindered the researcher from providing in-depth anthropological descriptions.

Another limitation of this research was ethical concerns. The ethical issues in ethnographic research can manifest in different ways, such as the researcher entering the informants’ world instead of inviting them to enter the researcher’s world. The informant observation technique is used in natural settings, often spanning weeks, months, or even years. In the ethnographic approach, limited information is provided to the informants or officials at the outset of data collection because more information is needed for the research. Another unique aspect is that the researcher may establish relatively close relationships with some informants, which can create obligations. The ethnographic report will describe the details of the informants and their activities, which may lead to their identification by those familiar with the situation being studied [45]. In this study, it was tried to consider the above items during the research.

## Conclusion

This critical ethnographic study in the ICU identified that ageism and lookism stereotypes emerged from seven main themes; youth-centric, negative ageism, age-friendliness, age-related priority, centered care for pediatric patients and families, appearance-centeredness, and a contradiction between belief and behavior. These themes shed light on the complex dynamics of parity in care in the ICU. The study’s findings underscore the prevalence of ageism and lookism stereotypes within the culture of ICU care, revealing the profound impact they have on patient treatment and interactions. These stereotypes can exacerbate healthcare disparities within the ICU, emphasizing the need for greater awareness and efforts to address these biases. As the healthcare community strives for equitable and patient-centered care, understanding and challenging these ingrained stereotypes is crucial to achieving this goal.

The key point in this study is that the influence of larger societies and cultures on smaller cultures cannot be ignored. Iranian society is currently undergoing a transition, where traditional values are evolving into values more aligned with the modern world. This transformation is accelerated through virtual platforms, publications, travel, and intercultural interactions of Iranians with other societies. Consequently, we encounter a society that places importance, value, and attention on youth. This societal perspective naturally extends to organizations and social institutions such as hospitals, impacting the behavior of healthcare personnel. Therefore, the attitudes of healthcare workers are shaped by societal perceptions of categories like age and beauty.

Additionally, the appreciation of beauty is a fundamental human inclination that resonates with many individuals. Humans are naturally drawn to and appreciate beauty. Therefore, it is not surprising that healthcare personnel may exhibit preferences for younger or more attractive patients. Conversely, it would be considered unnatural if they did not.

It is essential to underscore that healthcare professionals, like all individuals, may have varying attitudes toward patients. However, in terms of professional ethics, they are obligated to provide standard care to all patients without discrimination. The standard of care represents the minimum level of care expected. Therefore, as long as healthcare providers adhere to these standards for all patients, it is not ethically problematic if they exhibit preferences based on age or beauty. Nevertheless, continuous training and education are crucial to address and manage these biases effectively.

## Data Availability

The datasets generated and analyzed in this study are not publicly available because the data contain individual informants, but they are available from the corresponding author upon reasonable request.

## Abbreviations

ICU: Intensive Care Unit
